# Sef1-Regulated Iron Regulon Responds to Mitochondria-Dependent Iron–Sulfur Cluster Biosynthesis in *Candida albicans*

**DOI:** 10.3389/fmicb.2019.01528

**Published:** 2019-07-09

**Authors:** Shivani Ror, Sneh Lata Panwar

**Affiliations:** Yeast Molecular Genetics Laboratory, School of Life Sciences, Jawaharlal Nehru University, New Delhi, India

**Keywords:** iron homeostasis, mitochondria, iron–sulfur cluster, Sef1, *Candida albicans*, iron regulon, *FZO1*

## Abstract

Iron homeostasis mechanisms allow the prime commensal-pathogen *Candida albicans* to cope with the profound shift in iron levels in the mammalian host. The regulators, Sef1 and Sfu1 influence activation and repression of genes required for iron uptake and acquisition by inducing the expression of iron regulon genes in iron-deplete conditions and inactivating them in iron-replete condition. Our study for the first time shows that *C. albicans* coordinates the activation of the iron regulon with the mitochondrial use of iron for Fe–S cluster biosynthesis, a cellular process that is connected to cellular iron metabolism. We took advantage of a mutant defective in mitochondrial biogenesis (*fzo1*Δ/Δ) to assess the aforesaid link as this mutant exhibited sustained expression of the Sef1 iron regulon, signifying an iron-starved state in the mutant. Our analysis demonstrates that mitochondrion is pivotal for regulation of Fe–S cluster synthesis such that the disruption of this cellular process in *fzo1*Δ/Δ cells lead to excessive mitochondrial iron accumulation and reduced activity of the Fe–S cluster-containing enzyme aconitase. Sef1 responds to defective Fe–S cluster synthesis by regulated changes in its subcellular localization; it was retained in the nucleus resulting in the induced expression of the iron regulon. We predict that the mitochondrial Fe–S assembly generates a molecule that is critical for ensuring iron-responsive transcriptional activation of the Sef1 regulon. All told, our data marks Fe–S biogenesis as a mechanism that meshes cellular iron procurement with mitochondrial iron metabolism resulting in regulating the Sef1 regulon in *C. albicans*.

## Introduction

*Candida albicans* is a fungal commensal-pathogen that makes up the human microbiome and is the fourth leading cause of nosocomial bloodstream infections in immunocompromised human hosts (Brown et al., 2012). Iron is an essential micronutrient that influences the virulence and pathogenesis of *C. albicans* (Fourie et al., 2018). The gastrointestinal tract (GI), the commensal niche of this pathogen, remains enriched in iron due to non-absorption of dietary iron and may reach toxic levels in certain situations (McCance and Widdowson, 1938; Miret et al., 2003). On the contrary, in its pathogenic lifestyle *C. albicans* faces scarcity of iron in the blood (Martin et al., 1987). A number of studies show that *C. albicans* deploys sophisticated mechanisms to acquire iron from the host in iron-deficient environments, while safeguarding itself from iron toxicity in iron-replete regions. Thus, survival of *C. albicans* in the host is dependent on its ability to maintain intracellular iron homeostasis by meticulously regulating iron acquisition, utilization and storage (Chen et al., 2011).

**FIGURE 1:**
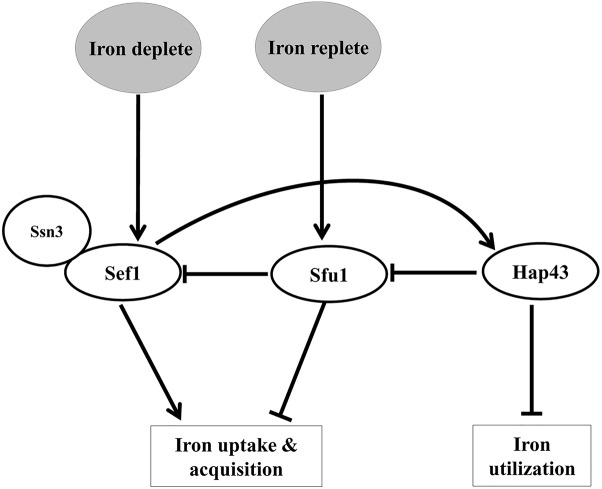
A scheme depicting the interplay of transcription factors in regulating iron homeostasis in *C. albicans*.

A tripartite system of transcription factors, Sef1 (Zn_2_Cys_6_ DNA-binding protein), Sfu1 (GATA factor), and Hap43 (CCAAT binding complex) regulate iron homeostasis in *C. albicans* (Figure 1) These transcription factors not only regulate each other but also control expression of iron uptake (Sef1, Sfu1) and iron utilization (Hap43) genes, collectively referred to as the iron regulon, thereby providing selective advantage to this fungus in environments with fluctuating iron levels (Chen et al., 2011). In iron-scarce situations, the increased expression of Sef1 and its activation by Ssn3 (Chen and Noble, 2012) allows the translocation of Sef1 to the nucleus resulting in the expression of genes that assist iron uptake and acquisition. Sef1 also induces Hap43 expression, which represses Sfu1 and iron-dependent processes (Chen et al., 2011). Iron sufficiency relieves the repression on Sfu1, enabling it to physically associate with Sef1, thus preventing nuclear localization of Sef1 and activation of iron-regulon genes (Chen and Noble, 2012).

In *Saccharomyces cerevisiae*, the control of cellular iron homeostasis is mediated by the transcription factor Aft1 (Rutherford et al., 2005). The role of mitochondria and their Fe–S cluster machinery in regulating various iron-dependent cellular processes in eukaryotes has been established (Lill and Mühlenhoff, 2006). The importance of Fe–S cluster biogenesis to cellular iron metabolism has been demonstrated by creating mutants that are defective in this cellular process. These mutants exhibit increases in mitochondrial iron and expression of iron regulon genes in iron sufficient conditions; a situation that otherwise is the signature of iron starved cells (Yamaguchi-Iwai et al., 1996; Kispal et al., 1999; Foury and Talibi, 2001; Bellí et al., 2004; Hausmann et al., 2008; Patil et al., 2013). Likewise, in *C. albicans*, the loss of *SSQ1* and *MGE1*, proteins involved in Fe–S cluster biogenesis leads to phenotypes similar to *S. cerevisiae* Fe–S cluster mutants (Demuyser et al., 2017; Dong et al., 2017). Considering that mitochondria serves as a site for the biogenesis of Fe–S clusters in eukaryotes, impairment in mitochondrial activity not only perturbs Fe–S cluster machinery but also results in the activation of the *S. cerevisiae* Aft1 and the iron regulon in iron-sufficient conditions (Chen et al., 2004; Patil et al., 2013). As disruption in Fe–S cluster synthesis is not associated with lowered cytosolic iron levels, Aft1 activation is suggested to be dependent on a product of the mitochondrial Fe–S biogenesis pathway rather than on cytosolic iron pools (Rutherford et al., 2005). Concordantly, compromising mitochondrial function, by deleting *FZO1*, in *C. albicans* also results in constitutive expression of the Sef1-regulated iron regulon in iron-replete conditions (Thomas et al., 2013).

In our previous study, we demonstrated that loss of *FZO1* affects mitochondrial morphology resulting in loss of mtDNA and perturbation of the mitochondrial membrane potential; thus generating cells with dysfunctional/defective mitochondria. *FZO1*, a GTPase and a component of the mitochondrial fusion machinery, is known to facilitate the biogenesis of mitochondria in yeast (Hermann et al., 1998; Rappaport et al., 1998; Mozdy and Shaw, 2003). Additionally, *fzo1*Δ/Δ cells exhibit increased susceptibility to azole antifungals and oxidants as well as altered ergosterol and phospholipid levels. A network model, based on the transcriptional analysis of the *fzo1*Δ/Δ cells, showed three distinct co-regulated gene clusters that are associated with specific biological processes, out of which the most striking subset of genes was the one that contained genes involved in iron homeostasis. Given the phenotypic overlap with respect to the altered expression of iron regulon between the mutants of the Fe–S cluster pathway and mitochondria, we posit a link between mitochondrial-dependent Fe–S cluster biogenesis and iron homeostasis in *C. albicans*. Although the significance of Fe–S cluster synthesis pathway in maintaining mitochondrial functions and iron homeostasis in *C. albicans* has been addressed (Dong et al., 2017), the question whether perturbations in the synthesis of Fe–S clusters are transduced to Sef1 for activating the iron regulon remain unanswered in this pathogenic fungus.

To systematically explore the importance of Fe–S cluster biogenesis for cellular iron homeostasis in *fzo1*Δ/Δ cells, we sought to evaluate the mitochondrial iron content and the activity of a Fe–S cluster containing-protein, aconitase. We report that aconitase activity is compromised in *fzo1*Δ/Δ cells and forced overexpression of key components (*ISU1*, *NFS1*, and *YFH1*) of the Fe–S biogenesis pathway in the mutant partially restore the activity of this enzyme. Sef1 activation and its retention in the nucleus underlies the increase in expression of the iron regulon in *fzo1*Δ/Δ cells, as the repression of this regulator is impaired in iron-replete conditions possibly due to lack of a functional Fe–S cluster biogenesis pathway. Thus, dysfunctional mitochondria not only perturb Fe–S cluster biogenesis but also affect the ability of Sef1 to sense the pools of cellular iron for regulating iron metabolism. Herein, we propose that iron sensing by Sef1 is linked to a signal that emanates from functional mitochondria in the form of Fe–S clusters. Collectively these data uncover a new regulatory pathway connecting the iron-dependent regulation of Sef1 activity to mitochondria-dependent Fe–S synthesis in *C. albicans*.

## Materials and Methods

### Media

The strains (Supplementary Table S1) were maintained as frozen stocks and propagated at 30°C on the following media. YEPD (yeast extract peptone dextrose; 10 mg/mL yeast extract, 20 mg/mL peptone, 20 mg/mL glucose, and 25 mg/mL agar) liquid medium and agar plates containing 200 μg/mL of nourseothricin (Werner Bioreagents) were used for selection of deletion mutants and nourseothricin resistant strains. To obtain nourseothricin sensitive derivatives of transformants, strains were grown in YPM (yeast extract peptone maltose; 10 mg/mL yeast extract, 20 mg/mL peptone, and 20 mg/mL maltose) for 8 h and plated on 25 μg/mL nourseothricin. *C. albicans* strains were routinely propagated in YEPD, also referred to as “iron-replete” medium. “Iron-depleted” medium is YEPD supplemented with one of the specific iron chelators, bathophenanthroline disulfonic acid (BPS). The following supplements, BPS (Sigma), Zymolase-20T (MP Biomedicals), Poly-L-lysine (Sigma), DAPI (4’,6-diamidino-2-phenylindole, Molecular Probes/Invitrogen) were added to the media/buffer at concentrations described.

### *In vitro* Growth Assays

Strains grown overnight in YEPD plate were diluted in 9 mg/mL saline solution to OD_600_ = 0.7. Then, 5 μL portions of four dilutions (5 × 10^3^–5 × 10^5^ cells) were spotted onto YEPD plates containing iron chelator, 500 μM BPS and BPS with added 100 μM FeCl_3_. Plates were photographed after 72 h at 30°C.

### Strain Construction

#### Construction of Deletion Cassettes for *FZO1*

*Candida albicans* mutants and complemented strains were created by standard two-step disruptions by using *SAT1*-flipper strategy as described (Thomas et al., 2013). Both alleles of *FZO1* were deleted in *sef1*Δ/Δ strain (Noble et al., 2010). Strains and plasmids used in the study are listed in Supplementary Tables S1, S2.

#### C-Terminal Myc Tagging

Myc-tagged allele of Sef1 (accession number XM_708348) (van het Hoog et al., 2007) was constructed by using pADH34 vector which contains a 13X Myc epitope tag preceding the *SAT1*-flipper cassette as previously described (Nobile et al., 2009). Primers used for amplification of cassette and detection for integration is listed in Supplementary Table S3. The polymerase chain reaction (PCR) amplicon obtained with primers *SEF1*mycFnostop and *SEF1*mycRUTR constitutes a 13X Myc epitope tag, *SAT1*-flipper cassette, 65-bp region homologous to *SEF1* ORF minus its stop codon on the 5′ end of the Myc tag and a 65-bp region homologous to *SEF1* UTR downstream of the stop codon on the 3′ end of the *SAT1*-flipper cassette (Gillum et al., 1984). This PCR product was transformed into SC5314 (wild type) and *fzo1*Δ/Δ. Correct integration of the C-terminal 13X Myc epitope tag and *SAT1*-flipper was verified by PCR using detection primers pairs *SEF1*upstreamcheckF and AHO300, *SEF1*downstreamcheckR and AHO301. The primer pairs *SEF1*downstreamcheckR and AHO302 were used to confirm the flipping out of the *SAT1*-flipper cassette. The 13X Myc epitope tag and the region of homology to the 3′ end of *SEF1* used for integration of the *SAT1*-flipper cassette was confirmed by sequencing the PCR product generated using primers *SEF1*upstreamcheckF and AHO283.

#### Overexpressor Strain Construction

(i) The *C. albicans* strains overexpressing *YFH1* (accession number XM_706449), *NFS1* (accession number XM_715279), and *ISU1* (accession number XM_716291) (Muzzey et al., 2013; Supplementary Table S1) were constructed using plasmid pCJN542 (Nobile et al., 2008). Primers amplify the *Ashbya gossypii TEF1* promoter, the *C. albicans NAT1* ORF, the *A. gossypii TEF1* terminator and *C. albicans TDH3* promoter with 100 bp of hanging homology to promoter region and 100 bp of hanging homology within the ORF from initiation codon of *YFH1*, *NFS1*, and *ISU1* (Supplementary Table S3). The transformation into wild type and *fzo1*Δ/Δ was done as described earlier and nourseothricin positive transformants were screened using detection primers listed in Supplementary Table S3. (ii) The *SFU1* overexpression strain in wild type (SN95) and *fzo1*Δ/Δ was created by using plasmid pSN141 as described by Chen and Noble (2012). pSN141 was engineered to contain (59–39): a PmeI site; 350–450 bp of sequence upstream of the *C. albicans LEU2* ORF; the *C. dubliniensis ARG4* gene (selectable marker); the *TDH3* promoter; the *SFU1* (accession number XM_718460) (Muzzey et al., 2013) ORF; 350–450 bp sequence downstream of the *LEU2* ORF; and a second PmeI restriction site. After digestion with PmeI, the plasmid was transformed into the strain SN95, generating SLP23 (Noble and Johnson, 2005). Correct integration of the insert in Arg^+^ transformants was verified by PCR, and overexpression of *SFU1* was confirmed by qPCR. *FZO1* gene was deleted in strain SLP23 by *SAT1*-flipper strategy and deletion was confirmed by Southern hybridization.

### Protein Extracts and Immunoblot Analysis

*Candida albicans* was grown at 30°C with starting OD_600_ of 0.4 and cells were harvested when OD_600_ reached 2.0. Culture was pelleted down (10,000 rpm; 4°C), washed with 1X PBS once and resuspended in 200 μL ice cold lysis buffer (50 mM Tris-HCl pH 7.5, 150 mM NaCl, 1 mM EDTA, 1% Triton 100X), 5 μL protease inhibitor cocktail, 10 μL phenylmethylsulfonyl fluoride (PMSF, 0.1 M stock) and 200 μL glass beads. Breaking was done for 1 min on cell breaker with intermittent chilling on ice three times, followed by centrifuging at 13,000 rpm to obtain a clear lysate, which was transferred into fresh tube. Total protein was determined using Bio-Basic BCA assay kit according to manufacturer’s instructions, and 100 μg of total protein was fractionated by SDS-PAGE using 10% Mini-Protean TGX gels (Bio-Rad). Fractionated proteins were transferred to nitrocellulose/PVDF membranes and blocked with 50 mg/mL BSA (in 1X PBS) for 2 h at room temperature. Membrane was incubated with 9B11 c-Myc primary antibody for overnight (Cell Signaling Technology) and then Horseradish peroxidase (HRP)-conjugated secondary antibody for 2 h (Bio-Rad). Blots were visualized with the ECL Plus Western blotting detection system (Biorad).

### Quantitative Real-Time PCR

*Candida albicans* strains were grown overnight in YEPD, sub-cultured from a starting OD_600_ of 0.3 in fresh YEPD and incubated at 30°C for 4 h. Total RNA, isolated using the RNeasy Mini Kit (Qiagen), was treated with DN*ase* I (Fermentas Life Sciences) to remove contaminating DNA. cDNA was synthesized with a RevertAid H Minus First Strand cDNA synthesis kit (Fermentas Life Sciences) according to the manufacturer’s protocol. Real-time PCRs were performed in a volume of 25 μL using the Thermo Scientific Maxima SYBR green mix in a 96-well plate. For the relative quantification of gene expression, the comparative threshold cycle (*C*_T_) method was used, where the fold change was determined as 2^-ΔΔ^*^C^*^T^. *ACT1* (accession number XM_019475182) (van het Hoog et al., 2007) was used as the internal control and the transcript level of the gene of interest was normalized to the *ACT1* (actin) levels.

### Immunofluorescence

Briefly, *C. albicans* strains carrying Sef1-Myc were grown at 30°C up to OD_600_ = 1.0, in “iron-replete” (YEPD, 1.23 μM basal Fe^3+^ concentration) and “iron-depleted” medium (YEPD supplemented with 500 μM BPS). As described by Inglis and Johnson (2002), cells were fixed with 4.5% formaldehyde for 1 h at room temperature and cell wall digestion was carried out with zymolase (1 mg/mL) for 30 min at 37°C with gentle shaking in total 500 μL SP (1.2 M sorbitol, 0.1 M potassium phosphate) buffer. Antibody hybridization were performed, 9B11 anti-c-Myc antibody (Mouse, Cell Signaling Technology) was used at a 1:300 dilution and detected with a 1:400 dilution of Cy2-conjugated secondary antibody (Jackson ImmunoResearch, 715-225-151). To visualize cell nuclei, cells were stained with 1 μg/mL DAPI (DAPI channel color was changed from blue to red). Slides were mounted with Fluoromount-G (Southern Biotechnology Associates, Inc.) and images were captured using 100X oil objective.

### Aconitase Activity Assay and Iron Estimation

#### Mitochondria Isolation

(i) *Preparation of crude mitochondria*. Cells were grown overnight and sub-cultured in 250 mL of YEPD broth overnight at 30°C with starting OD_600_ of 0.1. Cells were harvested at 5000 rpm for 10 min, washed one time each with 50 mL cold water and buffer A (50 mL, 1 M sorbitol, 10 mM MgCl_2_, 50 mM Tris-HCl, pH 7.8) supplemented with 30 mM dithiothreitol (DTT). As described by Li et al. (2011), 3 g of cells were suspended in buffer A for 15 min at room temperature with shaking (100 rpm), then collected and suspended in 15 mL of buffer A with 1 mM DTT containing 100 mg Zymolase 20T (Seikagaku Biobusiness, Inc.) per 15 g of pelleted cells. Cultures were kept for shaking (100 rpm) at 30°C for 60 min or until 90% of cells was converted into spheroplasts (as checked by light microscopy). Digestion was stopped by adding 15 mL of ice-cold buffer A and spheroplasts were washed twice with buffer A. Spheroplasts were suspended in 10 ml of cold buffer B (0.6 M mannitol, 1 mM EDTA, 5 mg/mL BSA, 1 mM PMSF, and 10 mM Tris-Cl, pH 7.4) and were broken mechanically using a Dounce homogenizer on an ice bath (15 times). Cell debris was removed by low speed centrifugation (1000 rpm for 10 min) in 15 mL falcon (chilled). The supernatant containing mitochondria was centrifuged at 10,000 rpm for 10 min and the pellet was washed twice with 10 mL of ice cold buffer C (0.6 M mannitol, 1 mM EDTA, 10 mg/mL BSA, 10 mM Tris-HCl, pH 7.0) in 15 mL falcon (chilled). Mitochondria were suspended in 500 μL of buffer D (0.6 M mannitol, 10 mM Tris-HCl, pH 7.0). (ii) *Purification of mitochondria*. Mitochondria purification was performed using a two-step gradient, 2 mL PB1 buffer containing 30% (v/v) Percoll TM was placed at the bottom of a centrifugation tube (Beckman, Inc) (PB1: 0.3 M mannitol, 10 mM TES, 1 mg/mL BSA. pH 7.5). Then, 2 mL of PB2 buffer containing 20% Percoll (v/v) was layered on the top of PB1 (PB2: 0.3 M sucrose, 10 mM TES, 1 mg/mL BSA, pH 7.5). The crude mitochondria preparation was layered on the top of the gradient which was then centrifuged at 40,000 × g for 45 min at 4°C. A whitish band (purified mitochondria) was collected from the interface of the Percoll gradient. Purified mitochondria were washed twice by centrifugation in 3 mL of PB1 buffer for 10 min at 3000 rpm (15 mL falcon).

#### Aconitase Activity Assay

Mitochondria were quantified using BCA protein assay kit (Bio Basic). Enzymatic activity of the mitochondrial Fe–S protein aconitase was measured as described earlier (Pierik et al., 2009). 950 μL of aconitase buffer was added to a quartz cuvette. Mitochondria were dissolved (50 μg) in 60 μL of mitochondria lysis buffer (MLB), mixed properly. Fifty microliters of this lysed mitochondria was added in quartz cuvette mixed well and absorbance was measured at 235 nm for 2 min. Δ𝜀_235__nm_ = 4950/M cm.

#### Iron Estimation

Purified mitochondria (100 μg of mitochondrial protein) were digested in 70% HNO_3_ by boiling for 2 min and then diluted to 30% HNO_3_. Iron content was determined using an inductively coupled plasma-mass spectrometry (ICP-MS).

## Results

### Dysfunctional Mitochondria Results in Mitochondrial Iron Accumulation

**FIGURE 2:**
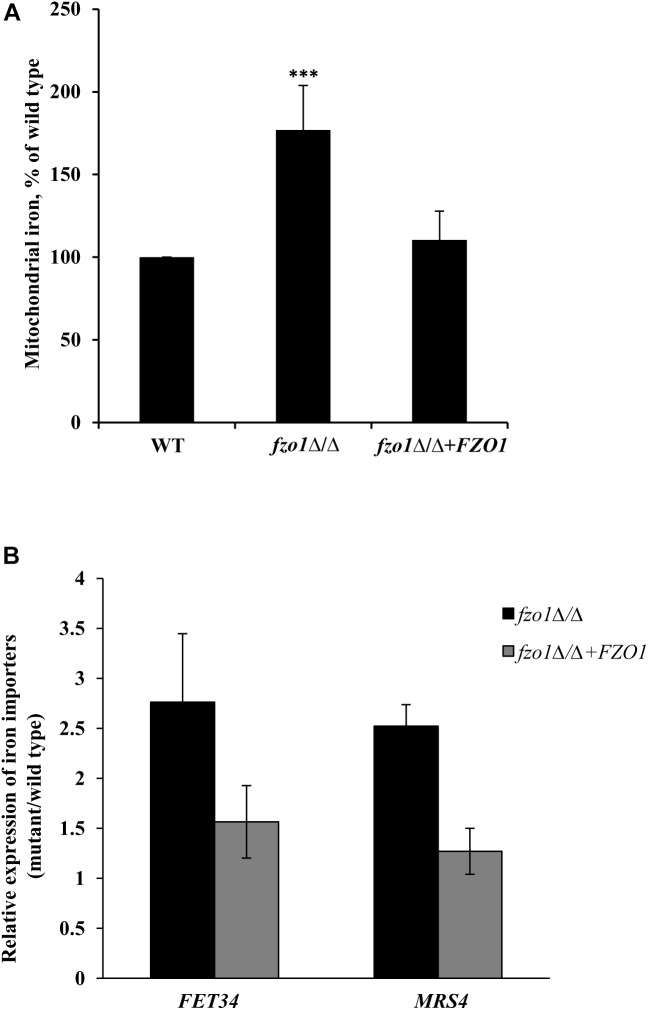
Dysfunctional mitochondria results in mitochondrial iron accumulation. **(A)** Mitochondrial iron overload in *fzo1*Δ/Δ. Cells were grown in YEPD at 30°C to the logarithmic phase and mitochondrial iron levels were determined by inductively coupled plasma-mass spectrometer. Data shown are mean ± SD (*n* = 9). **(B)** High expression of *FET34* and *MRS4* in *fzo1*Δ/Δ. The mRNA level of *FET34* and *MRS4* was quantified by qPCR in *fzo1*Δ/Δ relative to wild type. The *asterisk* indicates a significant difference relative to wild type, the two-tailed, unpaired *t*-test was used to determine the statistical relevance. ^∗∗∗^*P* < 0.001.

The constitutive high expression of the iron-regulon genes in *fzo1*Δ/Δ cells (Supplementary Table S4) even in iron-replete conditions (Thomas et al., 2013), is reminiscent of transcriptional changes observed during iron starvation and indicates toward the possibility of perturbed iron homeostasis in the mutant. We presumed that the constitutive expression of iron regulon genes in *fzo1*Δ/Δ cells might result in elevated iron levels in the mitochondria. To this end, we measured mitochondrial iron levels using ICP-MS in *fzo1*Δ/Δ cells. We show that mitochondrial iron levels in the *fzo1*Δ/Δ mutant were significantly increased by 77% relative to the wild type (Figure 2A). We expected that the induced expression of plasma membrane localized iron uptake system (*FET34*) and the mitochondrion membrane localized iron importer (*MRS4*) of the iron regulon might be supporting iron acquisition in *fzo1*Δ/Δ cells. In line with this, *fzo1*Δ/Δ cells exhibited twofold increase in the transcript level of *FET34* and *MRS4* (Figure 2B), supporting the accumulation of iron within the mitochondria. Altogether, this set of data suggests that, dysfunctional mitochondria may be the basis of mitochondrial iron overload and increase in expression of the iron regulon; indicators for perturbed iron homeostasis in *fzo1*Δ/Δ cells.

### *fzo1*Δ/Δ Displays Deficiencies in Fe–S Cluster Biogenesis

**FIGURE 3:**
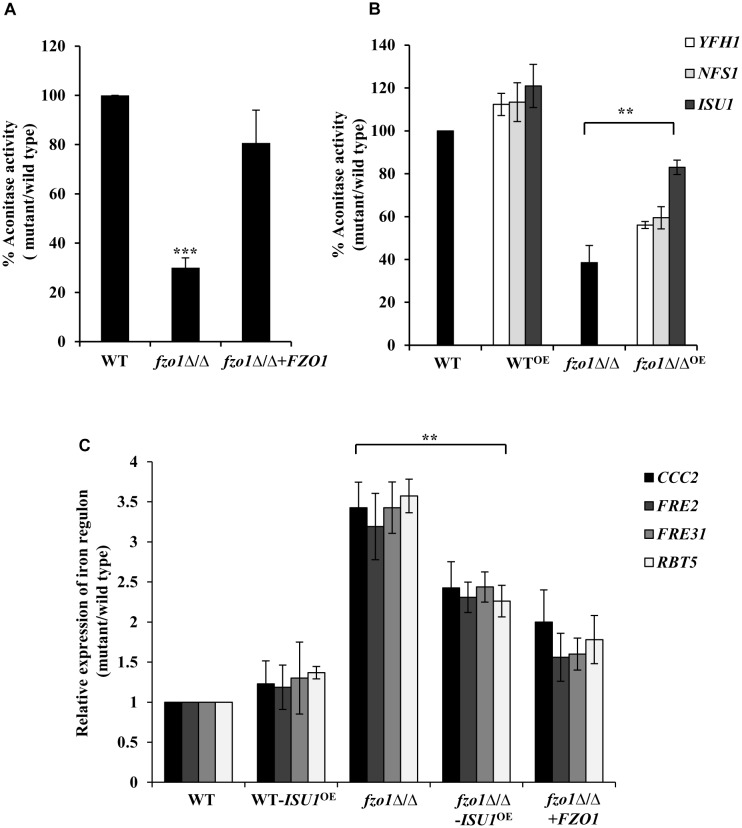
*fzo1*Δ/Δ displays deficiencies in Fe–S cluster biogenesis. **(A)** Decreased mitochondrial aconitase activity in *fzo1*Δ/Δ. Cells were grown in YEPD medium at 30°C and activity was assayed from purified mitochondria. Values are mean ± SD (*n* = 3). **(B)** Rescued aconitase activity in *fzo1*Δ/Δ-overexpressing strains of Fe–S biogenesis genes. WT^OE^ and *fzo1*Δ/Δ^OE^ are strains with overexpressing genes *YFH1*, *NFS1*, and *ISU1*. % Aconitase activity is calculated relative to wild type in panels **(A)** and **(B)**. **(C)** Overexpression of *ISU1* in *fzo1*Δ/Δ cells partially rescue upregulated expression of iron regulon genes. Indicated strains were grown in YEPD to logarithmic phase at 30°C and mRNA levels of indicated genes was quantified by qPCR. Relative transcript level was calculated by 2^-ΔΔ^*^C^*^T^, normalized to *ACT1* (endogenous control). Values are mean ± SD (*n* = 3) and are derived from three independent RNA preparations. The *asterisk* indicates a significant difference relative to *fzo1*Δ/Δ in panels **(B)** and **(C)**. Two-tailed, unpaired *t*-test was used to determine the statistical relevance. ^∗∗^*P* < 0.01; ^∗∗∗^*P* < 0.001.

Given that the activation of the iron regulon genes is dependent on the utilization of mitochondrial iron for the synthesis of Fe–S clusters (Garland et al., 1999; Chen et al., 2002; Hausmann et al., 2008), we reasoned that perturbed Fe–S cluster biogenesis in defective mitochondria may be the basis of iron homeostasis defect in *fzo1*Δ/Δ cells. To assess this, we measured the activity of the 4Fe–4S containing mitochondrial enzyme aconitase from purified mitochondrial extracts of wild-type, *fzo1*Δ/Δ and *FZO1* revertant strains grown in standard conditions. Aconitase activity in *fzo1*Δ/Δ cells was significantly decreased by 70% relative to wild type, whereas the activity of this enzyme was restored to wild type levels (80%) in the revertant strain (Figure 3A and Supplementary Table S5).

Furthermore, if absence of *FZO1* decreases aconitase activity because of defect in Fe–S biogenesis, then we assumed that overexpression of proteins involved in this process may compensate for (i) the decreased aconitase activity and (ii) the increased expression of iron regulon genes, in the mutant. The central components of the mitochondria-localized Fe–S cluster machinery include cysteine desulphurase (Nfs1), iron-chaperone (Yfh1), and Isu1/2 that serves as scaffold for *de novo* synthesis of Fe–S cluster (Lill et al., 2012). Hence, we created strains in which the endogenous promoter of *YFH1*, *NFS1*, and *ISU1* genes was replaced by the constitutively active *TDH3* promoter in wild type and *fzo1*Δ/Δ cells. We validated the expression of the transcripts of the selected genes in all the overexpressing strains (data not shown). Thereafter, aconitase activity was measured in wild type and *fzo1*Δ/Δ cells overexpressing *YFH1*, *NFS1*, and *ISU1* (*YFH1^OE^*, *NFS1^OE^*, and *ISU1^OE^*) (Supplementary Table S5). WT-*YFH1^OE^*, WT-*NFS1^OE^*, and WT-*ISU1^OE^* resulted in a 12, 13, and 21% increase in aconitase activity, respectively, compared to wild type (Figure 3B). Interestingly, the overexpressing strain *fzo1*Δ/Δ-*ISU1^OE^* exhibited 44% increase while *fzo1*Δ/Δ-*YFH1^OE^* and *fzo1*Δ/Δ-*NFS1^OE^* showed 18 and 21% increase in aconitase activity, respectively, compared to their parent strain (Figure 3B). This result suggests that the activity of aconitase, a Fe–S enzyme is affected in cells with dysfunctional mitochondria and that forced overexpression of genes involved in Fe–S biogenesis is able to partially compensate for the loss of aconitase activity in the *fzo1*Δ/Δ cells.

Next, we determined the expression levels of *CCC2*, *FRE2*, *FRE31*, and *RBT5* (iron regulon genes) in two independent clones of *fzo1*Δ/Δ-*ISU1^OE^*. We show that the expression level of the iron regulon genes in the *fzo1*Δ/Δ-*ISU1^OE^* strain was reduced similar to the reconstituted strain (ranged between 1.5 and 2.4-fold) when compared to *fzo1*Δ/Δ cells (Figure 3C), suggestive of partial repression of iron regulon upon forced overexpression of *ISU1*. Therefore, we surmise that insufficient mitochondrial activity results in defects in Fe–S synthesis, which in turn causes the induction of the iron regulon in *C. albicans*. Together, these results forge a link between the induction of iron regulon and mitochondrial Fe–S cluster synthesis in *C. albicans*.

### Induction of *SEF1* Is the Basis of Activated Iron Regulon in *fzo1*Δ/Δ

**FIGURE 4:**
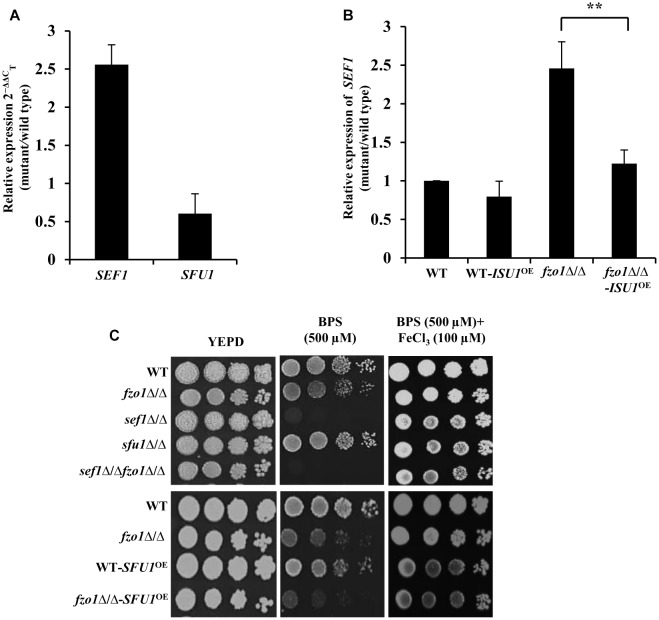
Induction of *SEF1* is the basis of activated iron regulon in *fzo1*Δ/Δ cells. **(A)** High expression of *SEF1* in *fzo1*Δ/Δ in iron replete condition. The mRNA level of *SEF1* and *SFU1* was quantified by qPCR in *fzo1*Δ/Δ relative to wild type. **(B)** Partial restoration of *SEF1* in *fzo1*Δ/Δ-*ISU1*^OE^ strain. qPCR–based expression analysis *SEF1* was done in *fzo1*Δ/Δ and *fzo1*Δ/Δ-*ISU1*^OE^. In panel **(A)** and **(B)** strains were grown in iron-replete medium and expression was normalized to internal control *ACT1.* Values are reported as fold change in expression over wild type. Data shown are mean ± SD (*n* = 3). The *asterisk* indicates a significant difference relative to *fzo1*Δ/Δ. A two-tailed, unpaired *t*-test was used to determine the statistical relevance. ^∗∗^*P* < 0.01. **(C)** For phenotypic comparison *C. albicans* strains were spotted on iron-replete (standard), iron-deplete (with iron chelators, BPS) and iron-supplemented medium (FeCl_3_ added in low-iron condition). Fivefold dilution were made and 5 μL of each dilution series was applied to solid test media, followed by incubation at 30°C for 72 h.

Transcriptome analysis of *fzo1*Δ/Δ cells in iron-replete condition showed a total of 27 differentially regulated genes that are annotated as genes involved in iron homeostasis in Candida genome database (CGD; Thomas et al., 2013). Out of the 27 genes, 16 genes are shown to be regulated by the transcription factors, Sef1, Sfu1, and Hap43 in *C. albicans* (Supplementary Table S4), pointing to the involvement of these transcription factors in regulating iron homeostasis in the mutant. As most of the Sef1-controlled iron regulon genes were upregulated, we evaluated the expression of this regulator in *fzo1*Δ/Δ cells. qPCR analysis performed in iron-replete condition confirmed significantly higher transcript levels of *SEF1* (2.5-fold) with no significant change in *SFU1* expression in the *fzo1*Δ/Δ cells, compared to wild type (Figure 4A). Furthermore, we argued that if the induced expression of *SEF1* requires the synthesis of the mitochondrial Fe–S cluster, then overexpressing *ISU1* in the *fzo1*Δ/Δ cells should repress the transcript levels of this regulator; an outcome of rescuing defective Fe–S cluster synthesis. Concordantly, we observed a 1.2-fold reduction in *SEF1* expression in *fzo1*Δ/Δ-*ISU1*^OE^, compared to the parent strain, affirming our proposition (Figure 4B).

The *fzo1*Δ/Δ mutant exhibited wild type susceptibility in iron-deplete medium (YEPD+BPS) (Figure 4C). We show that deletion of *FZO1* in *sef1*Δ/Δ background abrogated the growth of *sef1*Δ/Δ*fzo1*Δ/Δ cells in iron-deplete medium, compared to the parent strains (Figure 4C), suggesting that Sef1, in compliance with its role in wild type cells (Chen et al., 2011) also facilitates adaptation of cells with dysfunctional mitochondria to low iron stress. The specificity of the Sef1-dependent phenotype in iron-deplete medium was confirmed in *fzo1*Δ/Δ cells by (i) addition of FeCl_3_ to the BPS-treated medium and (ii) overexpressing Sfu1 in wild type and *fzo1*Δ/Δ cells. The growth defect of *sef1*Δ/Δ and *sef1*Δ/Δ*fzo1*Δ/Δ was reversed by FeCl_3_ addition to iron-deplete medium (Figure 4C). Next, we tested the ability of the WT-*SFU1*^OE^ and *fzo1*Δ/Δ*-SFU1^OE^* cells to adapt to low iron stress. Increased transcript level of *SFU1* was confirmed by qPCR in WT-*SFU1*^OE^ and *fzo1*Δ/Δ*-SFU1^OE^* cells (data not shown). In wild type cells, forced overexpression of *SFU1* (WT-*SFU1*^OE^) does not affect *SEF1* mRNA levels but leads to a decrease in Sef1 protein levels, suggesting a post-transcriptional role of Sfu1 in regulating Sef1 function. The *SFU1* overexpressing strain thus renders cells unable to tolerate low iron stress (Chen and Noble, 2012). Consistently, WT-*SFU1*^OE^ in this study also showed increased susceptibility to low iron stress condition, compared to the wild type (Figure 4C). We hypothesized that if Sef1 and Sfu1 interplay exists also in cells with dysfunctional mitochondria, then overexpressing *SFU1* in *fzo1*Δ/Δ should inhibit Sef1 activity resulting in diminished growth of the mutant in iron-deplete medium. In iron-deplete medium, the *fzo1*Δ/Δ-*SFU1*^OE^ strain showed a substantial growth defect compared to *fzo1*Δ/Δ cells, suggestive of the inhibitory effect of Sfu1 on Sef1 activity (Figure 4C). These results indicate that induction of *SEF1* is tied to mitochondrial activity and underlines the ability of the mutant to adapt to iron deficient conditions. In aggregate, these observations suggest that (i) inhibition of Sef1 activity in iron-replete conditions require functional mitochondria and (ii) Fe–S deficiency in *fzo1*Δ/Δ may be the basis of the loss of iron-responsive regulation of Sef1 in *C. albicans*.

### Sef1 Is Retained in the Nucleus in *fzo1*Δ/Δ

**FIGURE 5:**
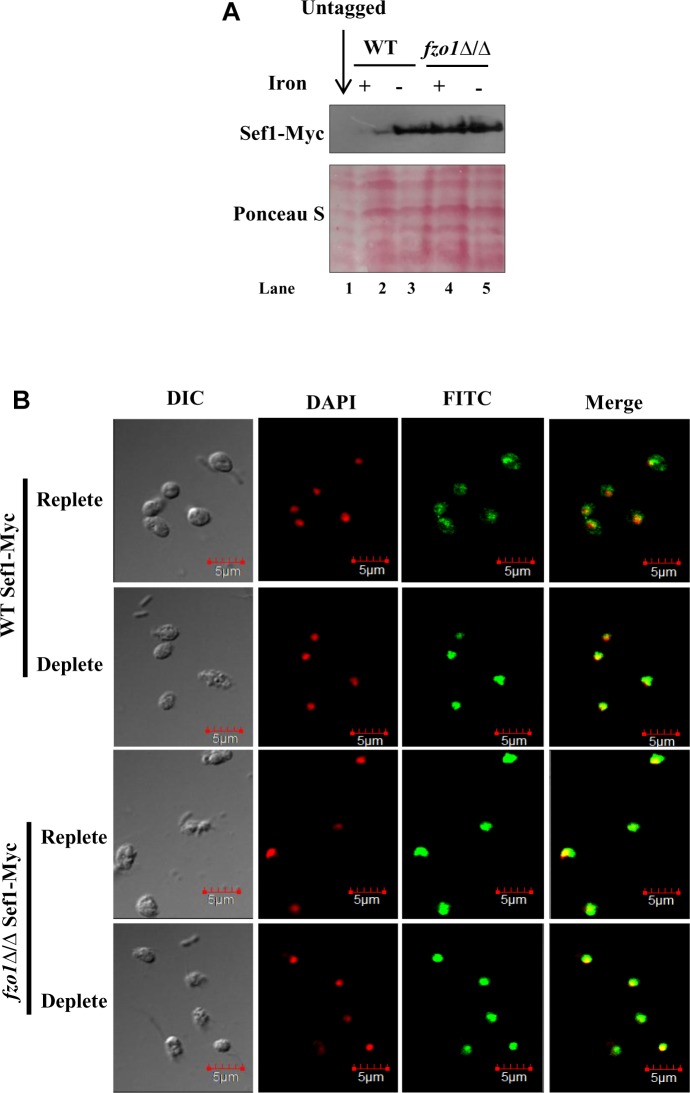
Sef1 is retained in the nucleus in *fzo1*Δ/Δ cells. **(A)** Immunoblot of Sef1-Myc with anti-Myc antibody in wild-type vs. *fzo1*Δ/Δ cells propagated under iron-replete (+; YEPD) or iron-deplete (–; YEPD + BPS) conditions. 100 μg of protein was loaded in each well. **(B)** Indirect immunofluorescence of Sef1-Myc in WT and *fzo1*Δ/Δ strain grown in iron-replete or iron-deplete medium. DIC represents phase images, FITC represents Sef1-Myc staining, DAPI represents DNA staining, and Merge represents the overlay of Sef1-Myc and DNA staining. Scale bar, 5 μm; all images were obtained at the same magnification.

To correlate the induced *SEF1* expression to its protein level and localization in *fzo1*Δ/Δ, we used an epitope-tagged version of Sef1 in which 13X-Myc epitope was fused in-frame at its C-terminus (Sef1-Myc). Immunoblotting with anti-Myc antibody was performed to measure the Sef1 protein levels. Previous reports demonstrate an inverse relationship between Sef1 protein levels and iron content in the growth medium (Chen and Noble, 2012). In line with this, the steady state level of Sef1-Myc was fivefold higher (quantified by ImageJ) in wild type cells grown in iron-deplete medium versus iron-replete medium (Figure 5A; lanes 2 and 3). Similarly, 6.6-fold high expression of Sef1-Myc was observed in *fzo1*Δ/Δ cells propagated in iron-deplete medium, compared to wild type in iron-replete medium (lanes 2 and 5). Strikingly, *fzo1*Δ/Δ cells in iron-replete condition exhibited sixfold higher Sef1 expression versus wild type cells in similar growth condition, indicating that the ability of Sef1 to sense cellular iron pools is impaired in cells with dysfunctional mitochondria (Figure 5A; lanes 4 and 2). The higher protein levels in the mutant correlated well with the induced *SEF1* transcripts (Figure 4A), reaffirming the iron-starved status of *fzo1*Δ/Δ cells even in iron sufficient condition.

Earlier studies have established that the localization of Sef1 varies as a function of iron wherein, Sef1 was localized primarily in the cytoplasm in iron-replete conditions and in the nucleus in iron-deplete conditions (Chen and Noble, 2012). To evaluate if Sef1 localization will vary as a function of mitochondrial activity, we performed indirect immunofluorescence microscopy on the wild type and *fzo1*Δ/Δ cells. As Sfu1 is known to be retained in the cytoplasm in iron-deplete medium, Sfu1-Myc expressing wild type strain was used as a control for cytoplasmic localization (Chen and Noble, 2012; Supplementary Figure S2). The localization of Sef1-Myc protein in wild type cells varied as a function of iron as it was localized in both cytoplasm and nucleus in iron-replete cells and redirected to the nucleus in iron-deplete cells (Figure 5B). By comparison, Sef1-Myc was not only primarily localized to the nucleus in *fzo1*Δ/Δ cells in iron-deplete medium but it was retained in the nucleus even in the presence of iron in the medium, indicating that the subcellular partitioning of Sef1 in response to iron availability in the medium is countermanded by insufficient mitochondrial activity. Collectively, these results demonstrate that the high expression and nuclear retention of Sef1 in *fzo1*Δ/Δ cells results in the constitutive activation of the iron regulon. We propose that inhibition of Sef1 activity under iron-replete conditions is dependent on a Fe–S cluster pathway signal furnished by functional mitochondria in this pathogenic fungus.

## Discussion

This study signifies the role of mitochondria in iron sensing which is crucial for harmonizing mitochondrial iron usage with cellular iron acquisition. Although the essentiality of mitochondria in regulating diverse cellular processes has been established, its role in regulating iron homeostasis in Sef1-dependent manner, by serving as the focal point of Fe–S biogenesis has not been addressed in *C. albicans*. Our use of *C. albicans* cells that harbor dysfunctional mitochondria (*fzo1*Δ/Δ) enabled us to establish, for the first time a link between mitochondria-dependent Fe–S cluster biosynthesis and iron sensing by the regulator, Sef1. Mitochondrial iron overload (Figure 2B) and abnormally elevated expression of iron regulon in growth medium containing sufficient iron denotes perturbed iron homeostasis and iron-starved status in *fzo1*Δ/Δ cells. This highlights that dysfunctional mitochondria impairs the iron responsiveness of iron regulon, such that the downregulation of the iron regulon genes in presence of sufficient iron is impeded.

**FIGURE 6:**
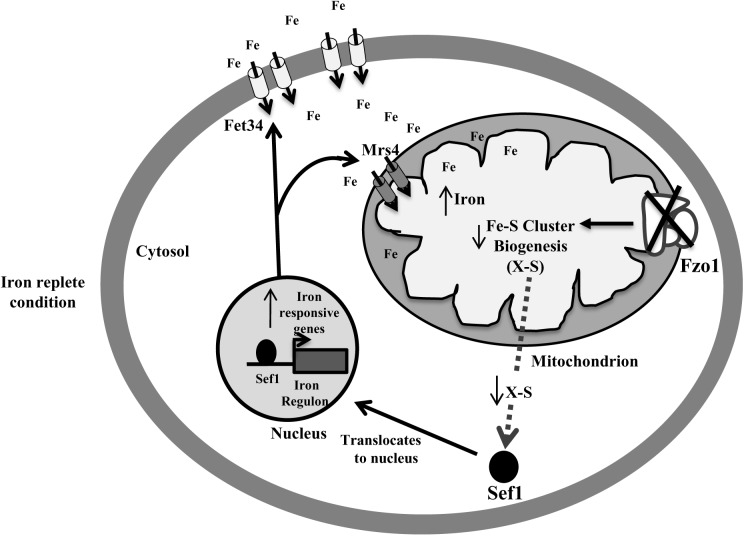
Activation of Sef1 is dependent on mitochondrial Fe–S biogenesis. Loss of *FZO1* leads to decreased Fe–S biogenesis, resulting in reduced activity of aconitase, a Fe–S cluster-containing protein. Decreased Fe–S biogenesis results in low Fe–S clusters and an unknown sulfur-containing compound (X-S) that may be exported into the cytosol. This unknown compound may be required for retaining Sef1 to the cytosol. Decreased level of compound X-S will signify iron starvation resulting in translocation of Sef1 to the nucleus. Consequentially, iron regulon genes including Fet34 and Mrs4 are activated leading to increased cytosolic iron acquisition followed by enhanced mitochondrial iron levels, in iron replete conditions.

Defective Fe–S biosynthesis due to insufficient mitochondrial activity, as is evident from decreased aconitase activity (Figure 3A), might be a plausible basis for the loss of iron responsiveness of iron regulon in *fzo1*Δ/Δ cells. While aconitase protein levels were not analyzed in the present study, the transcript levels of *ACO1* remain unchanged in the mutant (Supplementary Figure S1). We speculate that defective Fe–S cluster synthesis may be one of the basis for the decrease in aconitase activity in the mutant as reports in *S. cerevisiae* demonstrate that compromising Fe–S cluster synthesis does not affect aconitase protein levels/stability (Chen et al., 2002; Gelling et al., 2008). The partial restoration of aconitase activity and expression of iron regulon genes to wild type level, upon overexpressing the core components involved in Fe–S cluster synthesis, confirmed perturbed Fe–S biogenesis as the basis of deregulated iron homeostasis in *fzo1*Δ/Δ cells (Figure 3B,C). The transcriptional induction of *C. albicans FET34* in the mutant will lead to high iron acquisition, irrespective of the iron content in the exogenous medium (Figure 2A, 6). Consequentially, increased cytosolic assimilation of iron will result in increased import of iron in the mitochondria via *MRS4* resulting in mitochondrial iron overload in *fzo1*Δ/Δ cells (Figure 2A,B). Despite increased iron levels in the mitochondria, the mutant fails to synthesize Fe–S clusters due to defective Fe–S synthesis machinery. Shortage in the production of Fe–S clusters in *fzo1*Δ/Δ may provide a signal to the nucleus indicating insufficient bioavailable iron for Fe–S biosynthesis, thus generating a cellular iron starvation status in these cells. The cellular response to the iron starved status in *fzo1*Δ/Δ cells is the upregulation of the iron regulon, leading to mitochondrial iron overload, thus tying mitochondrial use of iron for Fe–S synthesis to cellular iron obtainment in *C. albicans* (Figure 6).

Despite them being tied to iron sensing, whether Sef1 and Sfu1 require additional regulatory inputs remains unanswered. Our findings established Sef1 as the key component of the iron homeostasis regulatory circuit in *fzo1*Δ/Δ cells based on (i) de-repression of iron regulon genes (*CCC2*, *FRE2*, *FRE31*, and *RBT5*) (Figure 3C) that are otherwise regulated by Sef1 in iron-deplete conditions, (ii) the inability of the *sef1*Δ/Δ*fzo1*Δ/Δ cells to adapt to iron deficiency in the medium (Figure 4C), and (iii) increased Sef1 levels in *fzo1*Δ/Δ during iron sufficiency (Figure 4A). Thereafter, based on the partial restoration of the *SEF1* transcript level to wild type levels upon forced overexpression of *ISU1* in *fzo1*Δ/Δ (Figure 4B), prompted us to suggest that iron sensing by this regulator depends on a signal from mitochondria and particularly from mitochondrial Fe–S cluster biogenesis in *C. albicans*. The iron-dependent localization of Sef1 was also affected in *fzo1*Δ/Δ cells; Sef1 was constitutively retained in the nucleus in iron-sufficient conditions (Figure 5B), arguing that mitochondrial-dependent Fe–S synthesis is a determinant of Sef1 localization. Although, iron regulation in yeast and higher eukaryotes differ substantially (Craig and Marszalek, 2002; Rouault and Tong, 2005; Lill and Mühlenhoff, 2006), the regulatory role of Fe–S biogenesis system is almost conserved in vertebrates, as mice or humans depleted of Abcb7 (involved in export of mitochondrial synthesized Fe–S compound) and patients suffering with low levels of frataxin (allosteric activator of the Fe–S cluster synthesizing enzyme cysteine desulfurase, *NFS1*) display iron accumulation in affected tissues (Pondarré et al., 2006; Popescu et al., 2007). Interestingly, precedence for Fe–S regulation of iron regulon expression is demonstrated by *S. cerevisiae* Aft1 that senses the status of Fe–S cluster production and export for regulating its own expression and localization (Yamaguchi-Iwai et al., 1995, 1996; Chen et al., 2004). Likewise, the iron sensing regulators HapX and SreA of *Aspergillus fumigatus* sense Fe–S cluster biogenesis to maintain iron homeostasis (Misslinger et al., 2018). This key molecule may be a Fe–S cluster-containing protein or a by-product of Fe–S biogenesis produced along with Fe–S clusters, which might be sensed by Sef1 or by a protein that associates with Sef1 for its activity (Figure 6). Aft1 of *S. cerevisiae* contains a C*X*C motif that if mutated (C293F) might hinder Aft1 from binding to Fe–S, resulting in a constitutively active allele, indicating the potential of this motif to be involved in directly binding to Fe–S cluster (Yamaguchi-Iwai et al., 1995; Chen et al., 2004). Sef1 is devoid of this motif but the possibility that this regulator may bind to another Fe–S cluster-containing protein for iron sensing can explain the dependence of *C. albicans* iron regulon on Fe–S cluster biosynthesis. Identification of the type of regulatory signal transduced from the mitochondria to Sef1 will decipher the mechanism underlying the cross-talk between mitochondrial Fe–S cluster biogenesis pathway and iron responsiveness of Sef1.

Many important questions pertaining to how iron regulates the expression and cellular localization of Sef1 in *C. albicans* remain to be elucidated. The questions include whether iron sensing by Sef1 exclusively depends on a signal originating from the mitochondrial Fe–S cluster synthesis or the cytosolic Fe–S assembly (CIA) also is a key in this process. Another question that arises is whether cellular iron status in *C. albicans* is involved in regulating the import or the export of Sef1 into the nucleus. The CIA is shown to be dispensable for iron sensing in at least *S. cerevisiae* and *A. fumigatus* (Chen et al., 2004; Lill et al., 2012; Misslinger et al., 2018). Deleting components of the CIA will address the relevance of this assembly process for iron sensing in *C. albicans*. Iron starved environments in the mammalian host serve as location markers for pathogens, signaling their entry into the host by triggering the expression of virulence genes. Given the established role of Sef1 in coupling iron acquisition with the expression of genes required for virulence, calibrating the levels of Sef1 is requisite in this pathogenic fungus. This is achieved by subjecting Sef1 to a series of post-transcriptional regulations (Chen and Noble, 2012). Disruption of these regulatory events affects the pathogenesis of *C. albicans* in a mammalian model of disseminated infection (Chen et al., 2011). Here, we add-on to the regulatory inputs in the form of mitochondrial-dependent Fe–S cluster biogenesis pathway that Sef1 perceives to activate the iron regulon for iron acquisition in the commensal-pathogen *C. albicans*. Multiple levels of regulation will assist in adjusting the activity of Sef1, thus empowering *C. albicans* to gauge and inhabit iron scarce and surfeit niches of the mammalian host.

## Data Availability

Publicly available datasets were analyzed in this study. This data can be found here: https://www.ncbi.nlm.nih.gov/geo/query/acc.cgi?acc=GSE46003.

## Author Contributions

SLP and SR planned the experiments and wrote the manuscript. SR performed the experiments and analyzed the data.

## Conflict of Interest Statement

The authors declare that the research was conducted in the absence of any commercial or financial relationships that could be construed as a potential conflict of interest.

## Supplementary Material

FIGURE S1 Expression of *ACO1* in *fzo1*Δ/Δ in iron replete condition. The mRNA level of *ACO1* was quantified by qPCR in *fzo1*Δ/Δ and *fzo1*Δ/Δ+*FZO1* relative to wild type. *ACT1* was used as internal control and data shown are mean ± SD (*n* = 3).

FIGURE S2 Localization of Sef1 and Sfu1-Myc in iron deplete condition. Shown images are indirect immunofluorescence of Sef1-Myc and Sfu1-Myc expressed in wild type, in iron-deplete (YEPD + BPS) condition. DIC represents phase images, FITC represents Sef1-Myc staining, DAPI represents DNA staining, and Merge represents the overlay of Sef1-Myc and DNA staining. Scale bar, 5 μm; all images were obtained at the same magnification.

TABLE S1 *Candida albicans* strains used in this study.

TABLE S2 Plasmids used in the study.

TABLE S3 Oligonucleotides used in this study.

TABLE S4 Differentially regulated genes associated with iron uptake/acquisition in *fzo1*Δ/Δ and regulated by transcription factors Sef1, Sfu1, and Hap43.

TABLE S5 Specific aconitase activity of strains used in Figure 3A,B.
