# Supplementary figures and images for: Sef1-Regulated Iron Regulon Responds to Mitochondria-Dependent Iron–Sulfur Cluster Biosynthesis in Candida albicans
Source: Front Microbiol. 2019 Jul 9;10:1528. doi: 10.3389/fmicb.2019.01528 (PMC6630100; doi:10.3389/fmicb.2019.01528)

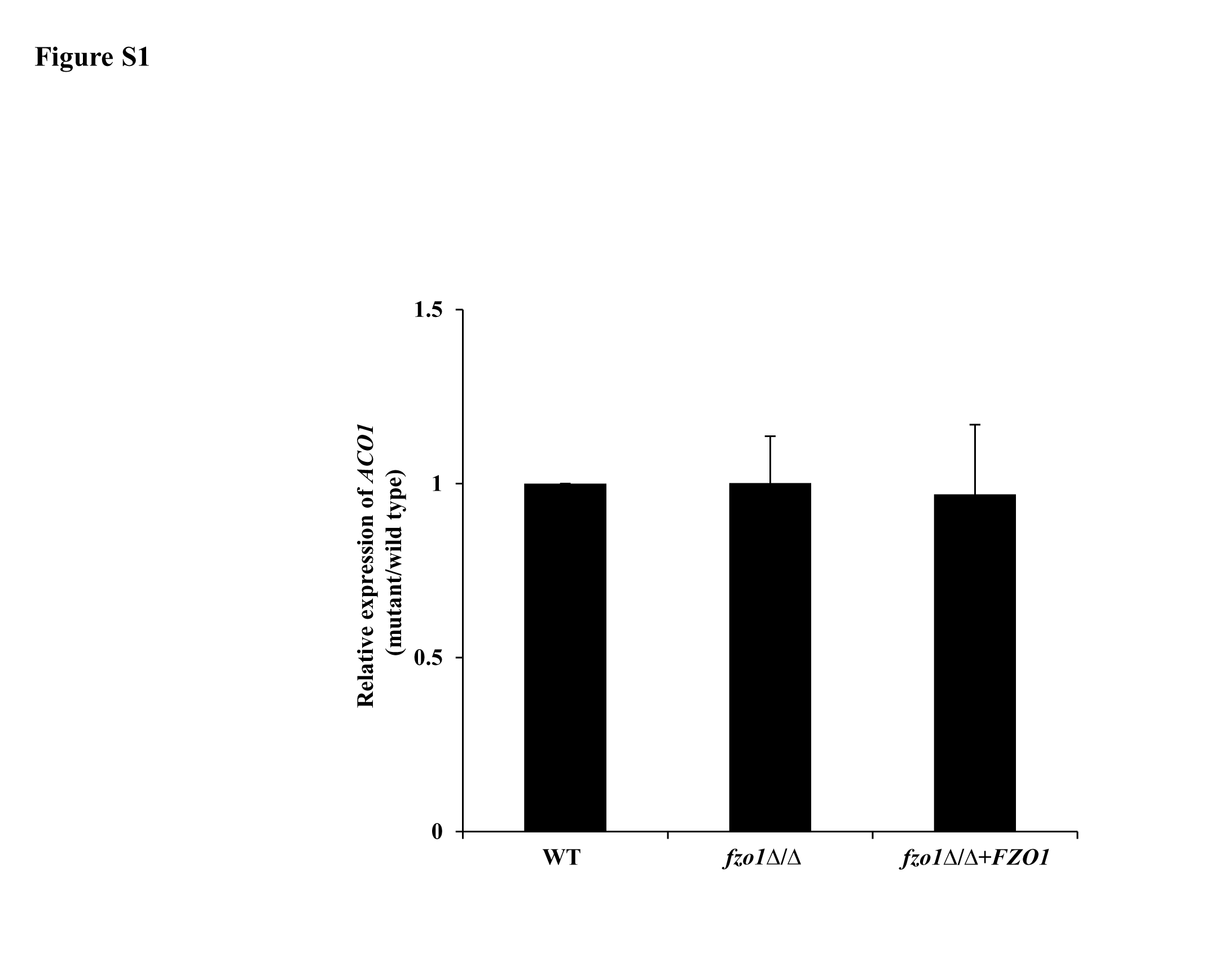

Supplement: FIGURE S1 — Expression of ACO1 in fzo1Δ/Δ in iron replete condition. The mRNA level of ACO1 was quantified by qPCR in fzo1Δ/Δ and fzo1Δ/Δ+FZO1 relative to wild type. ACT1 was used as internal control and data shown are mean ± SD (n = 3). [file Image_1.tif]

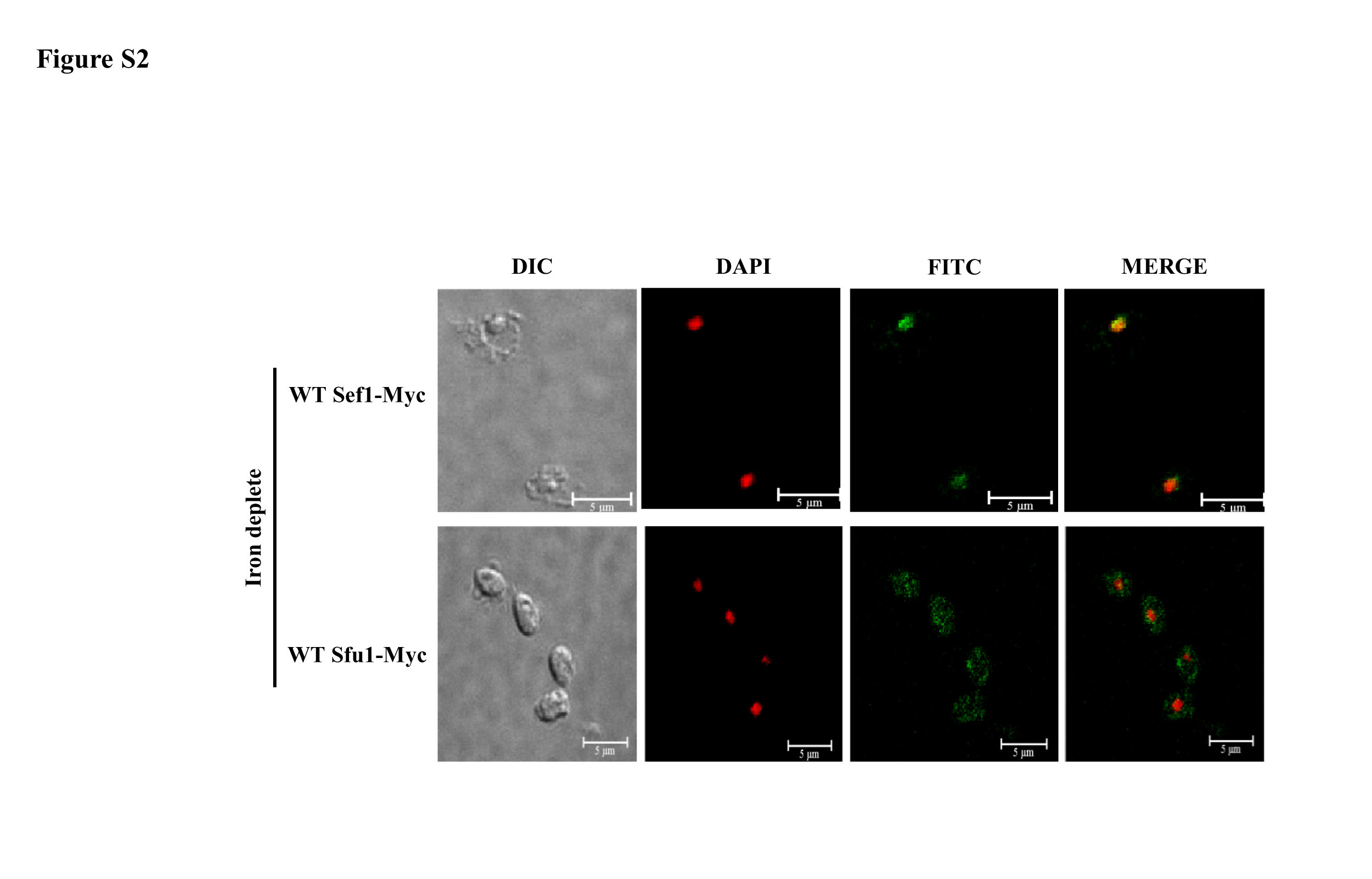

Supplement: FIGURE S2 — Localization of Sef1 and Sfu1-Myc in iron deplete condition. Shown images are indirect immunofluorescence of Sef1-Myc and Sfu1-Myc expressed in wild type, in iron-deplete (YEPD + BPS) condition. DIC represents phase images, FITC represents Sef1-Myc staining, DAPI represents DNA staining, and Merge represents the overlay of Sef1-Myc and DNA staining. Scale bar, 5 μm; all images were obtained at the same magnification. [file Image_2.tif]
