# Supplementary material for: Sef1-Regulated Iron Regulon Responds to Mitochondria-Dependent Iron–Sulfur Cluster Biosynthesis in Candida albicans
Source: Front Microbiol. 2019 Jul 9;10:1528. doi: 10.3389/fmicb.2019.01528 (PMC6630100; doi:10.3389/fmicb.2019.01528)
Supplement: TABLE S1 — Candida albicans strains used in this study. [file Table_1.docx]

**Table S1. *C. albicans* strains used in this study.**

C.d. *ARG4* denotes *Candida dubliniensis* *ARG4*, C.d. *HIS1* denotes *Candida dubliniensis HIS1*, and C.m. *LEU2* denotes *Candida maltosa LEU2*.

| **Strains** | **Parent** | **Genotype** | **Source** |
| --- | --- | --- | --- |
| SC5314 |  | Wild type | Gillum et al., 1984 |
| ET13 | SC5314 | *fzo1∆::FRT*/ *fzo1∆::FRT* | Thomas et al., 2013 |
| ET14 | ET13 | *fzo1∆::FRT*/*fzo1∆::FZO1-SAT1* | Thomas et al., 2013 |
| SN250 |  | *leu2Δ::C.m.LEU2/leu2Δ::C.d.HIS1, his1Δ/his1Δ, arg4Δ/arg4Δ, leu2*∆*/leu2Δ ura3Δ/URA3, iro1Δ/IRO1* | Noble et al., 2010 |
| SN95 | SN76 | *ura3::imm434::URA3/ura3iro1IRO1/iro1his1his1arg4/arg4* | Noble et al., 2005 |
| SN330 | SN250 | *sef1Δ::C.m.LEU2/sef1Δ::C.d.HIS1, his1Δ/his1Δ, arg4Δ/arg4Δ, leu2Δ/leu2Δ, ura3Δ/URA3, iro1Δ/IRO1* | Noble et al., 2010 |
| SN515 | SN250 | *sfu1Δ::C.m.LEU2/sfu1Δ::C.d.HIS1, his1Δ/his1Δ, arg4Δ/arg4Δ, leu2∆/leu2Δ, ura3Δ/URA3, iro1Δ/IRO1* | Noble et al., 2010 |
| SLP18 | SN330 | *FZO1*/*fzo1*∆::*SAT1*-FLIP | This study |
| SLP19 | SLP18 | *FZO1*/*fzo1*∆::*FRT* | This study |
| SLP20 | SLP19 | *fzo1∆::SAT1-*FLIP*/fzo1∆::FRT* | This study |
| SLP21 | SLP20 | *fzo1∆::FRT/fzo1∆::FRT* | This study |
| SLP22 | SLP21 | *fzo1∆::FRT/fzo1∆::FZO1-SAT1* | This study |
| SLP23 | SN95 | *SFU1-NAT1-TDH3p, ura3::imm434::URA3/ura3iro1IRO1/iro1his1his1arg4/arg4* | This study |
| SLP24 | SLP23 | *fzo1∆::FRT*/*fzo1∆::FRT* | This study |
| SN423 | SN250 | *SEF1-13xMyc/SEF1, his1Δ/his1Δ, arg4Δ/arg4Δ,leu2Δ::C.d.LEU2/leu2Δ::C.m.HIS1, ura3Δ/URA3, iro1Δ/IRO1* | Chen et al., 2011 |
| SLP25 | ET13 | *SEF1-13xMyc/SEF1, fzo1∆::FRT*/ *fzo1∆::FRT* | This study |
| SLP26 | SC5314 | *ISU1-NAT1-TDH3p* | This study |
| SLP27 | ET13 | *ISU1-NAT1-TDH3p, fzo1∆::FRT*/ *fzo1∆::FRT* | This study |
| SLP28 | SC5314 | *YFH1- NAT1-TDH3p* | This study |
| SLP29 | ET13 | *YFH1- NAT1-TDH3p, fzo1∆::FRT*/ *fzo1∆::FRT* | This study |
| SLP30 | SC5314 | *NFS1- NAT1-TDH3p* | This study |
| SLP31 | ET13 | *NFS1- NAT1-TDH3p, fzo1∆::FRT*/ *fzo1∆::FRT* | This study |
| SN646 | SN250 | *SFU1-13xMyc/SFU1, his1Δ/his1Δ, arg4Δ/arg4Δ, leu2*∆*::C.* *d. LEU2/leu2Δ::C. m. HIS1, ura3Δ/URA3, iro1Δ/IRO1* | Chen et al., 2011 |

**Table S2. Plasmids used in the study.**

| **Plasmid** | **Description** | **Source** |
| --- | --- | --- |
| pET1 | pSFS2B flanked 5’ and 3’ *FZO1* NCR for disruption of first allele of *FZO1* | Thomas et al., 2013 |
| pET2 | pSFS2B flanked 5’ *FZO1* NCR and 3’ *FZO1* ORF for disruption of second allele of *FZO1* | Thomas et al., 2013 |
| pET3 | *FZO1* reconstitution construct | Thomas et al., 2013 |
| pADH34 | C-terminal Myc tagging plasmid | Nobile et al., 2009 |
| pCJN542 | *NAT1*-*TDH3* promoter plasmid | Nobile et al., 2008 |
| pSN141 | *SFU1* overexpression plasmid | Chen and Noble, 2012 |

**Table S3. Oligonucleotides used in this study.**

| **Oligonucleotide** | **Description** | **Sequences (5’-3’)** |
| --- | --- | --- |
| *SEF1*-MycFnostop | To amplify 65-bp *SEF1* ORF myc tag region | TTAATAATGATAACCAAGATGACGACTTTTTGGGTTGGTTTGATGTTAATATGATGCAAGAGAAACGGATCCCCGGGTTAATTAACGG |
| *SEF1-*MycRUTR | To amplify 65-bp *SEF1* UTR myc tag region | ACTTATTCATTACAAAATCATATTAACATAATTACTAACTATTTACATTCTAATGAGGTAGAATCGGCGGCCGCTCTAGAACTAGTGGATC |
| DET-*SEF1-*Myctag UpF | To detect *SEF1*-myc construct integration | GGTATTCCCTTACCTATGCTT |
| DET-*SEF1-*Myctag DownR | To detect *SEF1-*myc construct integration | CACAAGGTGCCAGAATATACACA |
| AHO300 | To detect *SFE1* /*SFU1*-myc  construct integration | CCGTTAATTAACCCGGGGATC |
| AHO301 | To detect *SEF1* /*SFU1*-myc  construct integration | GGAACTTCAGATCCACTAGTTCTAGAGC |
| AHO302 | To detect *SEF1* /*SFU1*-myc construct integration | TCACTAGTGAATTCGCGCTCGAG |
| AHO283 | To detect *SEF1* /*SFU1*-myc construct  Integration and amplicon sequencing | GGCGGCCGCTCTAGAACTAGTGGATC |
| *YFH1*/OE/F | Forward primer for *YFH1*^OE^ | TAATAAATGTGCTCTATCTTTTAAAGACTGGTGTTGTTGATTAGCTTGGTCGTCCAAATCTTGTATCTCATCCGGTGGAACATATCCGTAATATTGTTGTTCAAGCTTGCCTCGTCCCC |
| *YFH1*/OE/R | Reverse primer for *YFH1*^OE^ | ATGTCTTGGTGATGAATCTGGTTTGAGGGTATATAAGTTGTCGAGTACAAGCTGGTTTGCTAAGGCATTTAGCTGAATTCAATGCAAATCTTTTGAACATATTTGAATTCAATTGTGATG |
| *YFH1*/DETp/F | Detection primer for *YFH1*^OE^ | CTTGAAGTCTTTCCTTTAATAAATGTGCT |
| *ISU1*/OE/F | Forward primer for *ISU1*^OE^ | TTTCTTTTTTCGGGGTTGTTTCTCCTTTCTTTTCTCCTCATTCCGTTTTTCTCATATTTTCTCTTTTTATTTTTGTAGATTTTACAGATAACGTATGGTCTCAAGCTTGCCTCGTCCCC |
| *ISU1*/OE/R | Reverse primer for *ISU1*^OE^ | TAGTGAATGTTGATGTTGGAATAGCTAATCTTGGAACAAGAACTCTTGATTGTTGAGTTAAAATTCTTCTTGTAGTAGGTAAAATCGTTCTGGTAAACATATTTGAATTCAATTGTGATG |
| *ISU1*/DETp/F | Detection primer for *ISU1*^OE^ | CACAAGATTAATATTATTTTCTTTTTTCG |
| *NFS1*/OE/F | Forward primer for *NFS1*^OE^ | TGGAATAGTACCATAATGAGAGAGATCCCATTCACGATGATTCAATTCCCATTATATGAATATTTAAAAGTGCAATGGCAACAAAATTTAAATAGTTTTATCAAGCTTGCCTCGTCCCC |
| *NFS1*/OE/R | Reverse primer for *NFS1*^OE^ | ATGAGCTGGTGGCCAATGGTTTAGGTAATGTAGTAACAAAGTTACGACTAGAAACTGTTTTCCCCAACCTGCCAGTCGTTTTGAAAATTGATTTATACATATTTGAATTCAATTGTGATG |
| *NFS1*/DETp/F | Detection primer for *NFS1*^OE^ | TTTGTATCGTGGTTGGAATAGTACCATA |
| Nat-OE-R-det2-CJN | Detection primer for overexpression | GAAACAACAACGAAACCAGC |
| *TDH3*p/DET/F | Detection of *SFU1* overexpression | TTAACCCTTGAAATTCCCTTC |
| *ISU1*/RT/F | Primer for qPCR | TGGGCTCGCTCAATAAAGAATC |
| *ISU1*/RT/R | Primer for qPCR | CACAAGCTGGAGCACCAACTAA |
| *YFH1*/RT/F | Primer for qPCR | TGCCTTAGCAAACCAGCTTGT |
| *YFH1*/RT/R | Primer for qPCR | GCAATGTCTTGGTGATGAATCTG |
| *NFS1*/RT/F | Primer for qPCR | GATGGGAAACCGACAAGGAA |
| *NFS1*/RT/R | Primer for qPCR | TCAGCGTTTATCACATCAGCAA |
| *MRS4*/RT/F | Primer for qPCR | TCAGTTGAAATAGACTACGAGGCAT |
| *MRS4*/RT/R | Primer for qPCR | ACATCACTGTGTGTTCCATTATACCA |
| *FET34*/RT/F | Primer for qPCR | TGTTGGTGGATTTGTGTCACAGT |
| *FET34*/RT/R | Primer for qPCR | CCCATCGACTTCAACGACAGT |
| *ACT1*/RT/F | Primer for qPCR | GAAGCCCAATCCAAAAGAGG |
| *ACT1*/RT/R | Primer for qPCR | CTTCTGGAGCAACTCTCAAT |
| *FRE2*/RT/ F | Primer for qPCR | AGGAAACAGACGGTGCACTTG |
| *FRE2*/RT/ R | Primer for qPCR | CAACTACTTAATCCAGACGACATT |
| *CCC2*/RT/ F | Primer for qPCR | ATGAAGTCACTGGCCATCCT |
| *CCC2*/RT/ R | Primer for qPCR | GGAATCGTCATACATTTAGA |
| *FRE31*/RT/F | Primer for qPCR | CACCAATGCAGCCAAACCA |
| *FRE31*/RT/R | Primer for qPCR | TTACTAAAGTTGACCGGTT |
| *RBT5*/RT/F | Primer for qPCR | GTGCAAGCCATTACCATGAGAGT |
| *RBT5*/RT/R | Primer for qPCR | ACGATCTATACGGCTATGACGAC |
| *SFU1*/RT/F | Primer for qPCR | GATGTAATGGTACTGGAGGATCGGC |
| *SFU1*/RT/R | Primer for qPCR | AGAGCTTTCATCTTCAGTAGTAGCG |
| *SEF1*/RT/F | Primer for qPCR | TCCACAACGATTGTCATATA |
| *SEF1*/RT/F | Primer for qPCR | GTTTAATGGCAAAGTAACGTCAC |
| *ACO1*/RT/F | Primer for qPCR | GGTTGAAATACAGAGGTCACTTG |
| *ACO1*/RT/R | Primer for qPCR | TATGACCAGCATCTCTGTAAGCA |

**Table S4. Differentially regulated genes associated with iron uptake/acquisition in *fzo1*∆/∆ and regulated by transcription factors Sef1, Sfu1 and Hap43.**

^a^ genes were downregulated in genome-wide expression profile of *sef1*∆/∆ (Chen et al., 2011).

^b^ genes validated in Thomas et al., 2013.

^c^ genes validated in this study.

^nd^ regulatory transcription factor is not determined.

| **Systematic**  **Name** | **Gene**  **Name** | **Predicted**  **Protein** | **Expression**  **(*fzo1*∆/∆**  **vs. WT)** | **Regulating Transcription Factor** |
| --- | --- | --- | --- | --- |
| orf19.5634 | *FRP1^a^* | Predicted ferric reductase | 22.01 | Sef1, Sfu1, Hap43 |
| orf19.1932 | *FRE5^b^*  (*CFL4*) | Ferric reductase | 22 | Sef1, Sfu1, Hap43 |
| orf19.4647 | *HAP32^a,b^*  (*HAP3*) | CCAAT-binding transcription factor that regulates respiration | 12.4 | Sef1, Sfu1, Hap43 |
| orf19.2179 | *SIT1^a,b^* | Transporter of ferrichrome siderophores | 7.9 | Sef1, Sfu1, Hap43 |
| orf19.4653 |  | Transcriptionally regulated by iron | 7.47 | nd |
| [orf19.1673](http://www.candidagenome.org/cgi-bin/locus.pl?locus=orf19.1673&seq_source=C.%20albicans%20SC5314%20Assembly%2021) | *PPT1* | Protein described as serine/threonine phosphatase | 6.86 | nd |
| orf19.5636 | *RBT5^a,b,c^* | GPI-anchored cell wall protein involved in hemoglobin utilization | 6.6 | Sef1, Sfu1, Hap43 |
| orf19.1930 | *FRE31^a,b,c^*  (*CFL5*) | Ferric reductase | 6.52 | Sef1, Sfu1, Hap43 |
| [orf19.5845](http://www.candidagenome.org/cgi-bin/locus.pl?locus=orf19.5845&seq_source=C.%20albicans%20SC5314%20Assembly%2021) | *RNR3* | Protein described as large subunit of ribonucleotide reductase | 4.42 | nd |
| orf19.4328 | *CCC2^a,b,c^* | Copper-transporting P-type ATPase required for iron assimilation | 3.1 | Sef1 |
| [orf19.4578](http://www.candidagenome.org/cgi-bin/locus.pl?locus=orf19.4578&seq_source=C.%20albicans%20SC5314%20Assembly%2021) | *CYT2* | Protein described as cytochrome c1 heme lyase | 2.51 | nd |
| orf19.1267.1 |  | Role in iron-sulfur cluster assembly | 2.3 | nd |
| orf19.1264 | *FRE2^a,b,c^*  (*CFL2*) | Putative oxidoreductase | 1.29 | Sef1, Sfu1, Hap43 |
| orf19.3527 | *CYT1* | Protein described as cytochrome c1 | -2.02 | Hap43 |
| [orf19.5893](http://www.candidagenome.org/cgi-bin/locus.pl?locus=orf19.5893&seq_source=C.%20albicans%20SC5314%20Assembly%2021) | *RIP1* | Protein described as subunit of ubiquinol cytochrome c-reductase | -2.04 | Hap43 |
| orf19.3358 | *LSC1* | Protein described as succinate-CoA ligase subunit | -2.08 | nd |
| orf19.1179 |  | Transcriptionally regulated by iron | -2.2 | Hap43 |
| [orf19.2013](http://www.candidagenome.org/cgi-bin/locus.pl?locus=orf19.2013&seq_source=C.%20albicans%20SC5314%20Assembly%2021) | *KAR2* | Similar to chaperones of Hsp70p family | -2.34 | nd |
| [orf19.4759](http://www.candidagenome.org/cgi-bin/locus.pl?locus=orf19.4759&seq_source=C.%20albicans%20SC5314%20Assembly%2021) | *COX5* | Protein described as cytochrome oxidase subunit V | -2.58 | Hap43 |
| orf19.675 |  | Cell wall protein | -2.6 | nd |
| [orf19.3822](http://www.candidagenome.org/cgi-bin/locus.pl?locus=orf19.3822&seq_source=C.%20albicans%20SC5314%20Assembly%2021) | *SCS7* | Protein described as ceramide hydroxylase | -2.72 | Hap43 |
| [orf19.6385](http://www.candidagenome.org/cgi-bin/locus.pl?locus=orf19.6385&seq_source=C.%20albicans%20SC5314%20Assembly%2021) | *ACO1* | Protein described as aconitase | -2.73 | Hap43 |
| orf19.465 | *IFF9* | Predicted GPI-linked cell-wall protein | -3.13 | nd |
| orf19.4747 | *HEM14* | Putative protoporphyrinogen oxidase involved in heme biosynthesis | -3.3 | Hap43 |
| [orf19.6321](http://www.candidagenome.org/cgi-bin/locus.pl?locus=orf19.6321&seq_source=C.%20albicans%20SC5314%20Assembly%2021) | *PGA48* | Putative GPI-anchored protein of unknown function | -3.45 | nd |
| orf19.2803 | *HEM13* | Coproporphyrinogen III oxidase | -3.6 | Hap43 |
| [orf19.4436](http://www.candidagenome.org/cgi-bin/locus.pl?locus=orf19.4436&seq_source=C.%20albicans%20SC5314%20Assembly%2021) | *GPX2* | Similar to glutathione peroxidase | -3.7 | nd |

**Table S5. Specific aconitase activity of strains used in Figures 3A and B.**

| **Strains** | **Specific aconitase activity (μmol/min/mg)** |
| --- | --- |
| Wild type | 1.58 |
| *fzo1*∆/∆ | 0.47 |
| *fzo1*∆/∆+*FZO1* | 1.27 |
| Wild type | 1.62 |
| WT*-YFH1^OE^* | 1.82 |
| WT*-NFS1^OE^* | 1.84 |
| WT*-ISU1^OE^* | 1.95 |
| *fzo1∆/∆* | 0.61 |
| *fzo1∆/∆-YFH1^OE^* | 0.91 |
| *fzo1∆/∆-NFS1^OE^* | 0.98 |
| *fzo1∆/∆-ISU1^OE^* | 1.34 |
